# Supplementary material for: Are carbon emissions trading and green financial instruments synergistic? -Comprehensive quantitative research based on content analysis
Source: PLoS One. 2024 Mar 7;19(3):e0298601. doi: 10.1371/journal.pone.0298601 (PMC10919601; doi:10.1371/journal.pone.0298601)
Supplement: S1 Data — (ZIP) [file pone.0298601.s001.zip › Data/地级市Z_F工作报告.zip!/║╙▒▒/╨╧╠¿╩╨/└┤╫╘╣1⁄2╓┌║┼í╛┬φ┐╦╩2╛▌═°í┐_2010.docx]

各位代表：

　　现在，我代表市人民政府，向大会作政府工作报告，请予审议，并请各位市政协委员和列席会议的同志提出意见。

　　一、2009年工作回顾

　　刚刚过去的2009年，是新世纪以来我市经济社会发展遇到困难最多、挑战最大的一年。面对国际金融危机的严重冲击，我们在省委、省政府的坚强领导和中共邢台市委的直接领导下，以深入开展学习实践科学发展观和干部作风建设年活动为动力，紧紧围绕保增长、调结构、扩内需、惠民生的主线，坚决贯彻落实中央和省应对危机的一揽子计划和政策措施，在迎接挑战中逆势而上，在攻坚克难中加快发展，较好地完成了市十三届人大二次会议确定的各项目标任务，保持了经济社会平稳较快发展的良好势头。

　　（一）经济总体回升向好。预计全市生产总值完成1060亿元，增长10%；全部财政收入完成110亿元，增长7.6%，其中地方一般预算收入完成43亿元，增长16.6%。全社会固定资产投资完成840.5亿元，其中城镇固定资产投资完成601.8亿元，均增长39.3%，为“十一五”以来最高增幅。141个项目列入省重点项目计划，数量列全省第3位，实现了县县都有省重点项目的历史性突破；200个市重点在建项目完成投资275亿元，增长19.6%；争取中央新增投资项目1510个、资金11.2亿元，超过前五年总和。建滔集团年产50万吨醋酸等39个项目竣工投产，金牛钾碱扩能等16个重大项目开工建设，宁晋盐矿开发等重大前期项目取得新进展。争取项目建设用地指标和急需开工项目用地指标均居全省前列。与中国建设银行河北分行、国家开发银行河北分行分别签订了总额度200亿元和90亿元的战略合作协议；全年各类金融机构新增人民币贷款190.8亿元，创历史最高纪录，是上年的4倍；市中小企业担保中心资本金达到3亿元，全年担保贷款超过10亿元。全年规模以上工业增加值完成382.5亿元，增长13.1%；社会消费品零售总额完成389.6亿元，增长17.9%。

　　（二）结构调整扎实推进。装备制造、新能源、煤盐化工、新型建材等产业加速扩张。以开发区为核心区域的邢台国家光伏高新技术产业化基地通过科技部认定，填补了我市没有国家级高新技术产业化基地的空白；晶龙集团高效单晶硅光伏发电项目列为国家“金太阳示范工程”。中钢邢机与华电集团签订了战略合作框架协议，金后盾与中国重汽合作年产5万辆重卡、邢钢不锈钢线材、御捷马电动车等项目积极推进。旭阳苯加氢一期、20万吨甲醇项目建成投产，宁晋盐化工园区整体规划通过环评。奎山集团加入冀东水泥集团，沙河迎新光伏玻璃项目建设顺利。羊绒、自行车、轴承等传统行业升级步伐加快。92个项目（企业）列入省重点产业调整振兴规划，数量全省第三。5家企业跻身中国企业500强，7家企业被认定为省创新型试点企业。新增中国驰名商标4个，总数由全省第7位上升到第4位。列入省“双三十”的县(市)和企业，均提前一年完成省三年考核减排目标。实施了20多个重大节能项目，形成了40万吨标准煤的节能能力。拆除钢铁企业高炉3座、水泥机立窑28座，关停小火电机组5台，取缔小造纸企业4家；市区拆除燃煤锅炉72台，出租车全部改用天然气，新增民用燃气1.3万户，发展集中供热面积251万平方米。4个园区列入省循环经济试点园区，建成16个污水处理厂、14个垃圾处理场。预计全年单位生产总值能耗下降5.78%，化学需氧量和二氧化硫排放量分别削减8%，均超额完成省定目标；市区空气质量二级及好于二级天数338天，比上年增加7天，其中一级天数68天，增加17天，空气质量首次达到国家二级标准，实现了历史性突破。

　　（三）城镇建设力度加大。预计全市城镇化率达到41%，同比提高1.5个百分点。市财政筹融资金47亿元，加大了城市建设力度。谋划实施了“一城五星”统筹措施，中心城区直管范围由200平方公里扩大到893平方公里，人口由70万增加到110万。对环市区五县（市）实施了按区管理，快速路建设全面启动，开通了市区到沙河市区、内丘县城、任县县城和邢台县皇寺镇、南和县贾宋镇的公交线路。交通投资完成36.4亿元，为历史之最。大广高速、邢汾高速、邢衡高速、石武高铁、邯（邢）黄铁路、邢和铁路等"东出西联、南北畅通"重大交通项目进展顺利，邢台机场民航复航筹建工作加快推进。95平方公里的城市建设用地实现了控规全覆盖；14个县（市、区）总规通过省、市审定。完成拆违拆迁1280万平方米，其中市区630万平方米，拆迁量居全省之首。全市共启动改造城中村67个，其中市区28个。郭守敬大道东段改建等9项国庆献礼工程完成，企业退市进园工作积极推进，启动了顺德路南延等10条断头路改造工程，泉南大街西延、中华大街西延等主路面通车，对14条主街道、85条小街小巷实施了综合治理改造。市图书馆、广电中心等基础设施工程开工建设，全民健身广场建成启用，新建了一批便民设施。市区绿化覆盖率、绿地率、人均公共绿地面积均超过国家园林城市标准。各县（市、区）大力推进县城建设，建成了一批精品、亮点工程。新改建农村公路1100多公里，100个省市级新民居示范村全部开工建设。

　　（四）农村经济稳定发展。认真落实强农惠农政策，发放各类补贴7.7亿元，增长16.6%。粮食总产80.7亿斤，连续六年丰收，总产、单产均创历史新高。畜牧、蔬菜、林果三大产业占农业总产值的比重达66%，同比提高8个百分点。引进了山东六和等一批农业产业化龙头企业，新上农业深加工和产业化项目172个，年销售收入5000万元以上的龙头企业达到50家，农业产业化经营率同比提高1个百分点。在全省率先完成了南水北调永久征地工作，地面附着物清理基本完成。13座水库除险加固工程顺利实施。全年输出农村富余劳动力10.8万人（次）。

　　（五）改革开放继续深化。市、县政府机构改革稳步推进，31户市属亏困工业企业基本完成改制或破产。组建了市投融资平台，整合“四资”65亿元。邢台海关获得国家批准，与韩国华城市建立了友好城市关系，引进了深圳科技工业园等一批重点项目，全年实际利用外资2.1亿美元。市开发区和清河开发区的扩区方案获省政府批准，沙河玻璃产业基地、隆尧东方食品城和宁晋西城工业区列入省级产业聚集区，县县都有了5平方公里以上的产业集中区。

　　（六）民生工程顺利实施。预计城镇居民人均可支配收入、农民人均纯收入分别实现13463元和4468元，增长11%和7%。全年城镇新增就业4万人，大中专毕业生就业2.8万人。新型农村合作医疗参合率91.6%，城镇居民医疗保险参保人数达到71万人，完成了省定目标；全市28万城乡低保对象的保障标准一步到位提高到全省平均水平，并建立了同步增长机制；“五保”对象集中供养率达到56%。筹资5亿元，安置国有工业和商贸破产、改制企业职工2.7万人。市区改造旧住宅小区4个、25万平方米，受益居民2777户；向1000户居民发放经济适用住房补贴共3000万元，各县（市、区）建成经济适用住房730套；全市发放廉租住房租赁补贴617万元，筹集实物配租住房累计达到10221套，居全省第2位，被省政府评为首批住房保障先进城市；农村教师住房竣工2153套；改建农村中小学88所。解决了68万农村人口的饮水安全问题，居全省第1位。

　　（七）社会事业协调发展。旭阳煤化工等3家工程技术研究中心纳入省管序列，结束了我市没有省级工程技术研究中心的历史；取得国内先进水平以上科技成果211项，居全省第4位，获省科技进步奖15项，数量创历史新高。学校、医院、乡镇卫生院、文化站等一批事关民生的社会事业项目开工建设或建成投用。市区和皇寺两个职教园区项目顺利实施；提前一年实现了基本普及高中段教育的目标；在义务教育学校实施绩效工资，促进了义务教育事业发展。农村计划生育家庭奖励落实率达到100%，被国家命名为“人口和计划生育综合改革示范市”。成功举办了中国·邢台首届太行山文化节，宣传我市形象的系列专题片在中央电视台《走遍中国》栏目播出。社区卫生服务街道覆盖率达到95%；采取积极措施，有效减轻了甲型H1N1流感等重大传染病疫情危害。在第十一届全运会上获得奖牌总数创历史之最，被省政府授予突出贡献奖，群众体育工作荣获全国先进称号。有效应对了几十年不遇的暴雨、暴风、暴雪等重大自然灾害。同时，双拥共建、广播电视、民族宗教、外事侨务、防震减灾、人防、气象、老龄、妇幼、残疾人、档案、地方志等各项社会事业也都取得了新成绩。

　　（八）平安创建成效显著。深入推进打黑除恶专项斗争，集中开展了涉黑恶线索大排查行动和“霹雳二号”集中收网统一行动，打掉了一批村霸、矿霸、沙霸等涉嫌黑恶犯罪团伙，依法惩处了一批犯罪分子，全年共破获各类刑事案件4582起，打掉犯罪团伙149个，查处治安案件6916起，有效震慑了犯罪，社会治安明显好转。圆满完成了国庆安保等各项工作任务，平安创建工作跨入全省先进行列。集中解决了一批群众反映强烈的热点问题和信访积案，化解了一批矛盾纠纷和不稳定隐患。安全生产和食品药品安全形势保持稳定。

　　（九）政府建设不断加强。干部作风建设年活动扎实开展，大力推进行政权力公开透明运行，市行政服务中心高效运转，县级行政服务中心全面启动运行，市、县长联系企业256家，发展环境进一步优化。压缩行政处罚自由裁量幅度70%以上，清理削减行政许可事项231项，审批环节和程序减少50%以上，审批时限压缩80%以上；在全省率先实现了网上审批和电子监察；行政许可事项集中办理率达到95.5%。房产登记办证在全省首创了“一手交房、一手交证”的新模式。坚持依法行政，自觉接受市人大及其常委会和市政协的监督，全年共办理市人大代表建议194件、市政协提案529件，按时办复率均为100%。主动听取各方面意见，接受社会各界的监督。

　　各位代表，过去一年是我市经济社会发展经受严峻考验的一年，也是各项事业继续向前推进、取得令人鼓舞新成就的一年，成绩来之不易，这是省委、省政府审时度势、坚强领导的结果，是中共邢台市委科学决策、积极应对的结果，是市人大、市政协和各民主党派大力支持、有效监督的结果，是全市人民众志成城、共克时艰的结果。在此，我代表市人民政府，向全市广大工人、农民、知识分子、各级干部和社会各阶层的建设者，向人大代表、政协委员，向各民主党派、工商联、无党派人士和人民团体，向各位老领导、老同志，向驻邢部队、武警官兵和政法干警，向中省直驻邢各单位，向所有关心和支持邢台发展的海内外朋友，致以崇高的敬意和衷心的感谢！

　　能够取得这样的成绩，主要靠两条，一是科学发展；二是实事求是。我们要认真坚持下去。同时，我们也清醒地看到，我市经济社会发展中仍然存在一些突出矛盾和问题。一是经济总体实力弱。与全省平均水平和周边地市比，经济总量小、发展质量差。全部财政收入和一般预算收入均排在全省后列，个别县财政收入出现了负增长，财政收支矛盾比较突出。大项目、高科技项目仍然较少。二是经济结构不优。三次产业比例不尽合理，农业产业化经营率低，工业主导产业规模小、产品科技含量低，服务业发展滞后。出口和消费对经济增长的拉动不足，投资作为主要动力的经济增长方式并未根本改变。三是城镇化水平低。城镇化率低于全国、全省平均水平，功能不完善，中心城市首位度低，带动辐射能力弱。四是社会事业欠账多。城乡居民收入增势趋缓，就业压力加大，教育、医疗、文化设施还不能充分满足群众需要。五是发展环境仍需进一步优化。个别政府部门和少数工作人员思想还不够解放，创新意识还不够强，在谋划思路时不够切合实际，目标还不够清晰；服务意识有待于提高，审批环节多、效率低下，吃拿卡要等现象仍然存在。对这些问题，我们一定高度重视，认真加以解决，决不辜负全市人民的殷切期望！

　　二、2010年工作目标要求和主要措施

　　今年是实施“十一五”规划的最后一年。做好经济社会发展的各项工作，对有效应对金融危机、保持经济平稳较快发展，为“十二五”规划启动实施奠定良好基础，具有十分重要的意义。

　　综观国际国内形势，经济社会发展环境将好于去年。国际金融市场渐趋稳定，世界经济有望恢复性增长。我国经济回升向好的基础逐步巩固，中央宏观经济政策的积极效应将进一步显现。我市正处于工业化、城镇化加速发展阶段，有着广阔的市场和丰富的资源，发展潜力很大。去年和今年新开工项目数量多、投资规模大，将对拉动经济增长形成有力支撑。“一城五星”建设步伐加快，区域竞争力正在形成。城镇面貌三年大变样进入决战之年，大规模城镇建设必将有力拉动投资和消费需求。经过近年来的快速发展，我市综合经济实力大幅提升，各项社会事业全面进步，全市上下谋求发展、摆脱落后的愿望和改革开放的意识不断增强，为我们加快发展积聚了强大动力。

　　但是我们也要充分认识到，全市经济社会发展仍然面临着复杂的形势和严峻的挑战。一是外部经济环境不确定不稳定因素很多，世界经济复苏将是一个缓慢复杂曲折的过程。二是进一步扩大需求存在较大制约，经济增长的内在动力依然不足。三是国家将加大对产能过剩行业的调控力度，过分依赖资源能源消耗的经济发展方式，再也难以为继，这对我们增投入、上项目提出了更高的要求。四是地区之间的竞争日趋激烈，各地都在抢抓机遇、竞相发展，我们面临的竞争压力更加突出。国家虽然继续实施积极的财政政策和适度宽松的货币政策，但也强调了将根据形势变化提高针对性和灵活性，我们必须认真研究新形势下的国家宏观政策，根据我市实际，采取有力措施，努力在转变发展方式上取得突破，保持全市经济平稳较快发展。

　　总之，面对复杂多变的发展形势，面对转瞬即逝的难得机遇，我们既要审时度势，增强机遇意识，充分看到有利条件和积极因素，始终坚定必胜信心；又要看到欠发达的现状仍没有根本改变，各种困难和不利因素依然较多，因此，我们要始终保持清醒头脑，采取积极有效应对措施，在新一轮竞争和发展中抢占先机、赢得主动，不断加快科学发展、富民强市的进程。

　　今年政府工作的总体要求是：认真贯彻党的十七大、十七届三中、四中全会和省、市委七届五次全会精神，以邓小平理论和“三个代表”重要思想为指导，全面落实科学发展观，按照中央和省、市经济工作会议的安排部署，围绕实现“科学发展、富民强市”总目标，坚持以增投入、上项目为根本举措，以提升经济实力、活力、竞争力为主攻方向，更加注重提高经济增长的质量和效益，更加注重推动经济发展方式转变和经济结构调整，更加注重推进改革开放和自主创新，更加注重改善民生、保持社会和谐稳定，更加注重统筹城乡发展，努力完成“十一五”规划的目标任务，实现经济社会又好又快发展。

　　今年经济社会发展的主要预期目标是：全市生产总值增长10%；全部财政收入增长11%，其中地方一般预算收入增长16%以上；全社会固定资产投资增长20%以上，其中城镇固定资产投资增长25%以上；规模以上工业增加值增长13%以上；出口总额增长8%左右；实际利用外资增长5%；社会消费品零售总额增长17%；城镇居民人均可支配收入增长10%，农民人均纯收入增长6%以上；单位生产总值能耗下降5%，化学需氧量和二氧化硫排放量分别削减3%和1%；城镇登记失业率控制在4.5%以内；人口自然增长率控制在8％。以内。

　　围绕上述要求和目标，我们将以“增投入、扩总量，调结构、促升级，惠民生、保稳定”为主线，坚持统筹城乡发展，重点抓好八个方面的工作。

　　（一）以项目建设为龙头，全力保持投资较快增长

　　把增投入、上项目作为关系邢台长远发展、打造未来产业优势的战略举措，继续强力推进“8222”投资促进计划，确保重点项目数量不少，投资强度不减，投资进度不慢，初步安排市级以上重点项目220个，年度投资280亿元。

　　一是加快重点项目建设。加快推进邢钢不锈钢线材、迎新光伏玻璃、晶辉高铁接触线等项目建设进度；力争沙河电厂、AP公司制氧、强大防爆电机、临西哈轴、平乡大行折叠自行车等工业项目和邯（邢）黄铁路、邢和铁路、邢衡高速、京港澳高速扩建等重大基础设施项目早日开工；加快中央新增投资项目建设，确保按时完成任务。

　　二是加大项目谋划力度。按照在建一批、开工一批、争取一批、储备一批的思路，重点围绕国家支持的新能源、节能环保、新材料等七个领域，在壮大主导产业、培育新兴产业、完善基础设施、改善民生等方面，谋划和储备一批带动力强、支撑作用大的项目，加强项目包装和对外合作，积极向上争跑，力争总投资亿元以上项目达到150项，有一批重大项目列入国家或省“十二五”专项规划。重点推进目前总投资5亿元以上的20个重大前期项目。继续做好下一步中央新增投资项目的筛选和争取工作。积极推进邢台机场民航复航筹建工作。

　　三是加强项目协调调度。继续实行市、县领导分包项目责任制和月通报、季调度、半年观摩、年终评比的工作机制。提高项目质量，坚决防止低水平重复建设和盲目扩张。严格实行项目投资责任制，加强项目管理，尤其是对有财政资金投入的项目，加强财政投资评审和监督，确保资金安全和工程质量。围绕项目建设，我们将千方百计争取上级项目资金和用地指标，争取金融信贷支持，争取战略投资者来邢投资，争取民间投资较快增长。

　　（二）以结构调整为主线，不断提高经济增长的质量和效益

　　以企业、产业的升级改造为抓手，推动结构调整迈出实质性步伐，不断提高经济增长的质量、效益和综合竞争力。

　　一是全力培育千亿产业。推进装备制造、新能源、煤盐化工和新型建材四个主导产业发展壮大，到2012年，四个产业工业增加值达到1000亿元以上，其中装备制造、新能源、煤盐化工三个产业的销售收入分别超过1000亿元，推动全市工业倍增发展。装备制造产业，重点围绕专用及通用设备、交通运输设备和基础配套产品等领域，积极推进冶金装备、重型汽车、机械配件等制造业发展。新能源产业，重点建设宁晋、开发区、沙河三大光伏产业基地，推进光伏产业全产业链发展；扩大太阳能在建筑领域中的应用，促进太阳能建筑一体化向纵深发展；支持中钢邢机、广进风电、华宇法兰等企业加快发展。煤盐化工产业，重点围绕甲醇、煤焦油、粗苯、矿盐深加工延伸产业链条，加快建设旭阳煤化工工业园、清修岗工业园、沙河煤化工工业园和宁晋盐化工工业园。促进煤化工骨干企业加快扩张提升，争创环保型领军企业，支持旭阳加强科技研发，争取利用两年时间，建成国家级煤化工工程技术研究中心和国家级煤化工循环经济产业园区。新型建材产业，重点发展玻璃深加工，加快建设沙河玻璃产业聚集区；支持发展新型干法水泥，积极推进水泥及水泥熟料基地建设；加快发展建筑新材料和新型墙材。

　　二是致力打造百亿企业。对综合实力强、技术水平高、发展潜力大的大型企业或成长型企业，加大支持力度，争取到2012年销售收入超100亿元的企业达到10家以上。支持有实力的企业开展整体并购、分散收购和强强联合，提高整体竞争实力。积极支持中小企业发展，市中小企业担保中心资本金力争达到5亿元，形成30亿元的担保能力。继续实施市、县长联系企业，做好政府采购优先购买本地产品、企业产需对接、要素供应和运行监测工作。在为招商引资引进企业搞好服务的同时，大力支持帮助现有企业做大做强。

　　三是改造提升传统行业。采用高新技术和先进适用技术改造传统优势产业，延长产业链条，提升产品技术含量和市场竞争力。钢铁行业，加大技术改造投入，不断提高装备水平，在稳定产能的基础上，抓品种、上质量，提高产品附加值。纺织服装行业，走花色多品种、产品深加工的路子，积极提升产品档次和技术工艺，提高产品市场竞争力。食品医药行业，积极开发休闲、绿色、环保、安全食品和饮品，优化食品生产企业布局，加快培育食品加工产业带和企业集群；着力提升医药生产和物流水平。自行车、轴承、玻璃、线缆等行业加快改造升级步伐。

　　四是培育发展新兴产业。着眼于占据产业发展高端和全面提升产业发展水平，积极引进关键技术和优质项目，培育发展电子信息、节能环保等高新技术产业和新兴产业，力争高新技术产业增加值增速高于规模以上工业增速。

　　五是加快园区建设步伐。按照“七通一平”标准，搞好园区基础设施建设。把项目进入园区作为申报省、市重点项目的必要条件，引导项目向园区聚集。力争年内所有市级园区通过环评，更多园区进入省级序列，努力把现有省级开发区和产业聚集区建成国家级开发区。加快市开发区新能源基地建设，确保签约项目和入驻项目双增长；对9个30万人口左右的县提倡“零乡镇”，促进园区向县城集中，人口向县城聚集。围绕打造百里太行工业经济带，加快提高官庄大孟工业园、德龙工业园等承载能力。

　　六是大力实施品牌战略。实施品牌建设“示范工程”，鼓励支持企业以高端、精品、专业化、深加工为方向，积极培育具有自主知识产权的产品和较强市场竞争力的品牌，争创驰名商标和名牌产品，提升企业知名度和美誉度。全年力争新增省以上知名品牌15个以上。

　　（三）以“三年大变样”为载体，扎实推进城镇化进程

　　增强现代城市意识和城市集约发展理念，对照省“三年大变样”考核指标体系，继续拆、突出建、强化管，规划引领、建管并举、以管促建，切实发挥城镇基础设施投资在增投入中的作用，力争全市城镇化率达到42.5%，水和空气环境质量得到有效改善。

　　一是深入实施“一城五星”发展战略。进一步提高“一城五星”规划建设水平，重点实施交通路网、市政设施和产业园区三大工程，优化城市布局，拓展城市发展空间。今年沙河、内丘养老保险、医疗保险、公教人员工资等达到市区水平，到2012年任县、南和达到市区标准，并逐步实现燃气、供热等市政公共设施一体化。加快连接“一城五星”路网和县城基础设施建设，年底实现“十分钟交通圈”。逐步明确“一城五星”功能分区，提高统筹发展水平，努力构建人口260万以上的大城市框架。

　　二是加快重点工程建设。积极推进环城高速、环城公路和城区路网建设改造，实施与石家庄红旗大街对接的南北大道一期工程；加快推进顺德路南延等工程，尽快打通一批断头路。启动改造旧城片区18个、城中村24个，对12家工业企业实施退市进园，用3-5年完成剩余的旧城片区、城中村、工业企业改造搬迁。加快推进七里河新区开发建设，启动西北部新区开发建设，加大火车站南片区、新世纪广场北片区、育英街道路及地道桥等重点项目推进力度，促进天一城商务中心、新亚购物中心改造等项目加快建设，努力建成一批精品和亮点工程。

　　三是加大拆违拆迁和城市容貌综合整治力度。拆违拆迁坚持规划先行，逐步由主要街道向小街小巷延伸，全年拆违拆迁800万平方米，其中市区350万平方米。启动河道治理和城区水系景观建设工程，加大对国省道两侧、出入市口等重点部位的环境综合整治力度，大力推进郭守敬大道精品绿化带、东三环景观带、百泉大道等建设整治工作。对县（市、区）城镇及市区中兴大街、新世纪广场周边、长途汽车站周边等，抓好以立面整修、牌匾整治、绿化提升、道路改造和线缆入地等为主要内容的景观整治，努力实现服务发展、适宜人居、环境优美的目标。

　　四是大力改善城镇居民生活条件。着眼于改善人居环境、完善城市功能，加强以绿化为重点的城市生态建设，以道路、桥梁、过街通道、停车场等为重点的市政设施和以学校、医院为重点的公用设施建设。加大新热源建设的谋划和推进力度，市区发展集中供热面积200万平方米，新增燃气用户1万户。围绕方便群众生活和店铺拆迁后商户安置，规划建设和培育一批便民市场。抓好城镇雨污分流工程建设，市区污水处理率达到88%以上，各县（市、区）达到70%以上。

　　五是积极推进城市精细化管理。进一步理顺城市管理体制，充分发挥三区一县在城市管理中的作用。加快数字化城管工程建设，建立城市管理信息指挥体系，推进网格化管理。进一步完善城市应急处置机制，实现规范化、常态化，提高城市管理水平。完善交通设施，推进公交提速；大力整顿市区交通秩序，科学管理、有效疏导，缓解交通压力。

　　六是提高城乡统筹发展水平。抓住邯（邢）黄铁路、邢和铁路、大广高速、邢汾高速、邢衡高速等项目建设机遇，谋划建设好引线和相关配套设施，建设县县之间的快速路，加快覆盖城乡的交通网络建设。加大县城和重点镇建设力度，放宽城镇户籍限制，使符合条件的农业人口逐步到城镇就业和落户。启动500个、重点抓好200个省市级新民居示范村建设，完善农村供水、供电、通信、道路、沼气、广播电视等基础设施，搞好村庄绿化，改善环境卫生，推进农村面貌改变。

　　（四）以节能减排为抓手，加快转变发展方式

　　今年是实现“十一五”节能减排目标的最后一年，我们将进一步加大工作力度，大力发展低碳经济，保护好生态环境，促进发展方式转变。

　　一是抓好省、市“双三十”示范工程。重点抓好列入省“双三十”的县(市)和企业的节能工作，确保按期或提前完成省三年考核目标。抓好列入市“双三十”的30个重点工业集中区和30家重点企业的节能和减排工作，不折不扣地完成既定目标。

　　二是加大落后产能淘汰力度。严格执行国家产业政策和省制定的淘汰落后产能工作计划，坚决淘汰钢铁、电力、建材等行业的高耗能、高污染落后产能，为结构调整腾出容量、腾出空间。

　　三是加快节能减排重点项目建设。重点抓好23个中央预算内投资的节能、节水、综合利用项目和12个节能奖励项目，争取早日投产达效。积极推进21个市级重点节能项目，抓好4个省级循环经济试点园区、4家省级循环经济试点企业和25个市级循环经济示范项目建设。市区新建太阳能一体化建筑60万平方米。加强太行山生态建设。

　　四是巩固环境综合整治成效。全部拆除市区具备集中供热条件的燃煤锅炉，其余改用清洁能源。加强对城市汽车尾气的治理，强化建筑施工、道路扬尘污染防治，市区空气质量二级及好于二级天数保持在330天以上，使牛城的天更蓝、空气更清新。

　　五是强化督导考核。落实目标考核制度，加强统计监测与信息反馈，对节能减排工作进展缓慢、逾期完不成目标任务的地方和企业，严格执行问责制和“一票否决制”。

　　（五）以农民增收为核心，认真做好“三农”工作

　　始终把“三农”工作作为重中之重，巩固和加强农业基础地位，促进农业增效、农民增收、农村繁荣。

　　一是重视发展粮食生产。继续落实和完善各项强农惠农政策，及时足额发放各类涉农补贴资金；落实好国家新增千亿斤粮食生产能力建设规划中我市承担的任务；加大对产粮大县的扶持力度，推进以良种培育为重点的农业科技创新，扩大优良品种种植面积，大力推广“两晚”、病虫害综合防治等实用技术，加快农业科技成果转化，确保全市粮食总产稳定在80亿斤以上。

　　二是加快调整优化农业结构。根据我市区域农业资源禀赋的现状，积极发展近郊农业、观光农业、生态农业、设施农业。在黑龙港平原重点发展生猪、蛋鸡、奶牛、肉禽养殖四大产业，加快规模化、标准化养殖基地建设。在环市区重点打造现代高效设施农业示范区，任县、南和大力发展设施蔬菜，建设市区蔬菜主要供应基地。在近郊丘陵区鼓励发展坑塘养殖，推广高产、高档水产养殖。在中西部丘陵地带重点利用好南水北调工程，优化弃土方案造地，整合太行山绿化工程等资金，大力发展薄皮核桃种植和林下养殖，力争今年增加面积10万亩，尽快形成百里薄皮核桃产业带。西部山区重点抓好苹果、板栗等优质干鲜果品种植加工基地建设。

　　三是提高农业产业化经营水平。以项目建设为载体，大力推进蛋鸡、奶业、食用菌、干鲜果品、方便面、淀粉加工等特色产业向规模化、集约化、专业化方向发展，加大对六和春蕾等龙头企业的帮扶力度，培育一批有市场竞争力和影响力的农产品品牌，新增年销售收入5000万元以上龙头企业5家，农业产业化经营率达到60%。

　　四是加强农业基础设施和服务体系建设。加强农田水利基本建设，年底前全部完成列入专项规划的大中型和重点小型水库除险加固工程；扎实推进南水北调工程，确保2月底前完成征迁安置工作。继续实施土地治理等农业开发项目，提高农业生产综合保障能力。加快发展农民专业合作组织，提高农业生产经营组织化程度。切实抓好动物防疫、质量检测、农业保险、防灾减灾等农业服务和安全体系建设；严厉打击制售假冒伪劣行为，整顿规范农资市场秩序，维护农民权益。

　　五是千方百计增加农民收入。引导资金、技术、人才、管理等要素向县域流动，壮大县域经济。开展好农村土地承包经营权流转和建设村级集体财富积累机制试点工作，推进征地制度改革，深化集体林权制度改革。加强农民技能培训，多渠道促进农村富余劳动力向城镇和非农产业转移，全年转移30万人（次）。

　　（六）以扩大消费为导向，大力发展服务业

　　一是大力发展现代物流业。抓住国家调整振兴物流业、河北省打造冀中南物流通道的机遇，着眼于正在形成的交通枢纽地位，谋划打造覆盖京津冀、连接晋鲁豫、辐射国内外的现代物流体系。整合资源、培育龙头，重点建设好望角、龙岗等综合型物流园区和粮食、医药、烟草等专业性物流中心，提升食品、轴承、玻璃、羊绒等特色市场的物流水平。

　　二是积极发展金融业。大力改善金融生态环境，增多做强金融市场主体，打造多层次的金融体系。促进城市商业银行、农村信用社、村镇银行、小额贷款公司等地方金融机构加快发展；完成市商业银行更名工作，促进市商行实现跨区域经营；积极引进域外金融实体在我市开展业务、设立机构；推进企业上市融资，支持河北建滔、龙星炭黑、中钢邢机等企业年内上市；鼓励国家工作人员为农户提供小额贷款担保，组织好农户间的联保，解决农村经济发展中贷款难的问题。

　　三是促进旅游业发展。以河北省发展邢台百里太行生态旅游为契机，围绕打造"太行明珠、守敬故里、魅力邢台"的城市旅游品牌，进一步完善旅游业总体规划。突出抓好旅游业项目建设，以邢汾高速公路为依托，建设西部游客综合服务中心。加大政策扶持和宣传推介力度，塑造龙头、打造精品，促进旅游业由单纯的观光游向观光与休闲度假游并重转变。

　　四是拓宽居民消费领域。扩大住房、汽车等大宗消费，发展商贸、家政、社区物业等服务性消费，开发文化网络、体育健身、健康的娱乐休闲等新的消费领域，依托城市主城区、产业集中区和交通枢纽，建设一批服务业集聚区。培育工业研发、信息服务等新兴服务业，大力发展文化产业。鼓励社会资本投入民生服务体系建设，发展便民利民的生活服务网点。深入实施“万村千乡市场工程”，加快建设“双百市场工程”，积极推进“农超对接”试点，落实好“家电下乡”等政策，培育农村消费热点。

　　（七）以改革开放为动力，努力活跃发展全局

　　一是深化重点领域改革。完成市、县政府机构改革任务，探索创新开发区、工业园区管理体制。抓好精减审批事项的落实，防止反弹。进一步发挥财政的职能作用，加强政府投融资平台建设；科学调整有关财政体制，支持市本级骨干企业加快发展，培植和壮大市本级财源，做大市本级财政；各级财政要强化综合治税，依法做到应收尽收，做大财政支出，优化收支结构。加快整合交通、粮油、物流等方面的国有优势资源，探索组建跨部门、跨行业、产权清晰、法人治理结构完善的集团公司；进一步完善经营性国有资产管理体制，努力实现国资监管的全覆盖；做好国有企业职工安置、破产改制企业资产处置和扫尾工作；加快供销社产权制度改革步伐，进一步完善农村现代流通网络。加大矿产资源整合力度。深化医药卫生、文化等体制改革。

　　二是放手发展民营经济。降低市场准入门槛，健全资金、土地、技术、人才、优惠政策等方面的服务体系，努力创造各类市场主体平等竞争发展的局面。进一步推动和鼓励自主创业，营造全民创业、增收致富、竞相发展的浓厚氛围，提高民营经济增加值在全市生产总值中的比重。

　　三是大力推进科技创新。加大对科技创新的支持力度，鼓励和推进企业自主创新，促进企业与高等院校、科研院所的技术合作，加快科技成果转化。支持企业加大科研投入，鼓励中小企业朝着“精、深、专、特”方向发展；实施百名企业家素质提升工程，积极引进技术人才，加强企业人才队伍建设。聚集和激活创新要素，建设创新型邢台。

　　四是加大招商引资力度。瞄准国内外行业龙头和领军企业，瞄准港、澳、台和长三角、珠三角等重点地区，精心组织招商活动，以“筑巢引凤”、“引凤还巢”等形式，提高招商针对性，确保招商实效。重点抓好投资500万美元以上的招商项目，提高外资到位率、合同履约率、项目开工率。继续下大力优化投资软环境，为客商搞好服务。

　　五是着力扩大对外贸易。加快邢台海关筹建工作，积极推进保税仓库、保税区、内陆港建设，加强与港口的合作，打通出海通道，提升我市经济外向度。优化出口商品结构，提高产品科技含量和附加值，转变外贸增长方式，对钢材、建材、机电、纺织服装等重点行业和企业，加强服务协调，努力扩大出口。

　　（八）以改善民生为根本，积极推进社会事业全面进步

　　围绕保障和改善民生，认真办好十个方面关系人民群众切身利益的实事、好事，促进经济社会协调发展。

　　一是完善社会保障体系。扩大社会保险覆盖面，将更多的中小企业就业人员、灵活就业人员、农民工纳入社会保险覆盖范围。切实抓好国家新型农村养老保险试点，进一步提高企业养老、失业和工伤保险待遇。稳步提高城镇职工、居民医保住院费用报销比例。完善新型农村合作医疗制度，每人每年财政补助标准提高到120元。

　　二是千方百计扩大就业。继续实施积极的就业政策，多渠道创造就业岗位；实施“百千万”人才工程，突出抓好大中专毕业生就业工作；实施万人上岗就业工程，积极开发公益性岗位，落实好各项优惠政策，重点解决困难大学生、零就业家庭、下岗困难职工的就业问题；实施城乡技能扶助计划，优先安置城镇拆迁失业人员、下岗职工和就业困难人员。鼓励有能力的人员自主创业、自谋职业，以创业促就业，城镇新增就业4万人，下岗再就业1.54万人。

　　三是大力帮扶困难群体。完善城乡低保水平与全省同步增长机制，稳步提高低保户待遇，加强对低保边缘群体的救助，实现动态管理下的应保尽保；加大“五保”供养工作力度，加快敬老院、光荣院、福利院“三院合一”工作；积极开展城乡医疗救助，对低保家庭中重病重残人员、高龄老人、儿童和缺乏劳动力家庭实施重点救助；大力发展社会福利和慈善事业；加大扶贫开发工作力度，基本完成第三批230个贫困村的整村推进任务；用两年时间基本解决农村饮水安全问题。

　　四是推进安居工程建设。加强房地产市场监管，增加中低价位、中小套型普通商品房供应，稳定住房价格。支持居民自住和改善性购房需求，利用3年左右时间，基本解决市区低收入家庭住房困难问题。将有稳定收入的城镇就业大中专毕业生、农民工逐步纳入保障性住房范围，力争筹集廉租住房1500套。加快棚户区改造步伐，完成拆迁20万平方米。加快农村教师住房建设，切实改善农村教师居住条件。

　　五是提高居民收入水平。认真落实国家有关政策，下大力提高城乡居民特别是低收入群众的收入水平。在落实义务教育学校绩效工资的同时，按照国家部署加快推进其他事业单位绩效工资的实施。适时提高公务员补贴、津贴标准。

　　六是优先发展教育事业。进一步加快高校建设步伐。大力发展职业教育，加快市区职教园区和皇寺职教园区建设，进一步提升中等职业教育办学水平。健全义务教育经费保障机制，推进义务教育均衡发展，逐步解决农民工子女免费接受义务教育问题。实施农村中小学校舍安全工程；坚持教育向园区集中、中学向县城和重点镇集中，小学向乡镇集中，加快农村中小学布局调整。加强教师培训，提高师资队伍素质。完善家庭经济困难学生资助体系，确保每一名家庭经济困难学生都得到有效资助。更加重视青少年道德和法律知识教育，为广大青少年健康成长创造良好环境。

　　七是提高医疗服务水平。进一步完善公共卫生设施，加强县级医疗机构、重点乡镇卫生院建设，90%县（市、区）的乡镇卫生院完成标准化、规范化建设；努力改善提高农村卫生室的设施条件和医疗水平，社区卫生服务街道覆盖率达到98%；建立健全城市卫生支援农村卫生事业长效机制；大力实施医疗惠民工程，减轻群众就医负担；加强甲型H1N1流感等重大疫病防控，提高突发公共卫生事件的应急处置能力。加强人口计生工作，做好我市第六次全国人口普查工作。

　　八是繁荣文化体育事业。积极推进文化设施建设，图书馆年底投入使用，博物馆下半年开工，积极推进乡镇综合文化站和农家书屋工程建设；实施文化精品工程，深入挖掘和整合本土文化资源，着力打造郭守敬、太行山、牛城等文化品牌；抓好文化信息资源共享、流动舞台车等重点文化工程，推动文化资源向农村和基层倾斜。在县（市、区）建设20条健身路径，为500个行政村安装健身器材，推动全民健身运动深入开展。

　　九是切实加强公共安全。健全和落实安全生产制度，强化安全生产监管责任，抓好高危行业、人员密集场所、道路交通等安全管理，有效防范和遏制重特大安全生产事故的发生。扎实开展食品安全监管长效机制建设年活动，强化政府监管责任和企业主体责任，完善监管网络，切实让人民群众买得放心、吃得安全。

　　十是维护社会和谐稳定。做好信访和矛盾调处工作，积极排查化解和妥善处理社会矛盾，认真解决征地拆迁、企业改制、环境污染、涉法涉诉等关系群众利益的突出问题。完善突发事件应急处置机制，有效预防和妥善处置各类突发事件。完善规划、加大投入，深入开展平安乡镇（街道）、平安社区（村庄）、平安单位（企业）创建活动，进一步加大对黑恶势力的打击力度，斩草除根、除恶务尽，力争用2-3年时间，将我市建成全省乃至全国最平安、最稳定地区之一。

　　重视做好编制“十二五”规划工作，认真开展双拥共建、广播电视、民族宗教、外事侨务、防震减灾、人防、气象、老龄、妇幼、残疾人、档案、地方志等各项工作，努力取得新成绩。

　　三、切实加强政府自身建设

　　发展的重任、竞争的态势、人民的意愿，对政府自身建设提出了更新、更高、更严的要求。我们将始终带着责任、使命和激情，为了邢台人民的利益，为了邢台的明天，做好每项工作，让邢台的发展一年更比一年强，人民生活一年更比一年好！

　　（一）坚持解放思想，创造性地开展工作。深化学习实践科学发展观活动成果，真正把科学发展的理念化作实实在在的行动，把科学发展的要求落实到每一项具体工作中。解放思想、与时俱进，把改革创新的精神贯穿到政府工作的全过程，用创新的思维想问题、办事情，用改革的办法解难题、促发展。进一步强化机遇意识，千方百计用好国家和省促进经济社会发展的一系列政策措施，抓住时机、乘势而上，以超常规的思路、超常规的举措推进各项重点工作的开展，不断取得新成绩。

　　（二）坚持学习研究，提高科学决策水平。加强对科学发展、市场经济和国家政策、法律法规的学习研究，提高政策理论水平，增强工作的前瞻性、预见性、针对性。敏锐把握和有效应对形势变化，及时了解各种新情况、新问题、新矛盾，善于抓住主要矛盾，有针对性地提出解决办法。广泛听取各方面意见建议，集思广益，集中民智，使决策更加科学。

　　（三）坚持转变作风，加大工作落实力度。巩固和运用好干部作风建设年活动成果，强化服务意识，提高行政效率，多讲怎么办，少讲不能办，积极主动为市场主体搞好服务，千方百计为人民群众解决实际困难，努力建设服务型政府。纠正行业不正之风，切实解决损害群众利益的突出问题。强化执行能力，确定目标任务，明确时限要求，每一项具体目标都要落实到具体单位、具体人，确保各项工作按照既定部署，一步一步、扎扎实实地落到实处。强化督导检查，盯住不落实的人，抓住不落实的事，严格执行问责制。

　　（四）坚持依法行政，规范行政执法行为。严格依照法定权限和程序履行职责，切实做到依法行政。严格执行人大及其常委会的决议、决定，自觉接受人大及其常委会的法律监督和工作监督；加强与人民政协的协商，自觉接受人民政协的民主监督；广泛听取各民主党派、工商联和无党派人士的意见和建议。认真办理人大代表建议和政协提案。深入推进政务公开，切实保障人民群众的知情权、参与权、表达权、监督权。强化执法监督，确保严格执法、公正执法、文明执法。加强行政复议工作。重视发挥新闻舆论和社会公众的监督作用。

　　（五）坚持廉洁自律，建设人民满意政府。以完善惩治和预防腐败体系为重点，切实加强反腐倡廉建设，严格落实廉政建设责任制，坚持用制度管权、管人、管事，确保权力规范运行、透明运行、阳光运行。始终牢记“两个务必”，严格遵守廉洁自律的规定和要求，勤勉尽责、秉公用权，堂堂正正做人，清清白白做官，干干净净做事。树立过紧日子的思想，厉行节约，勤俭办事，严格财政支出管理，严格公费出国出境管理，严格控制修建楼堂馆所，严格规范公务接待行为，坚决反对铺张浪费和奢靡之风，以实际行动和工作业绩，建设廉洁、务实、高效的政府，建设有所作为的政府，建设人民满意、人民信赖的政府！

　　各位代表！

　　回顾过去，我们豪情满怀；展望未来，我们信心百倍。我市正处在经济社会加快发展的关键时期，困难和挑战考验着我们，机遇和期待召唤着我们，责任和使命激励着我们。让我们紧密团结在以胡锦涛同志为总书记的党中央周围，在省委、省政府和市委的坚强领导下，在市人大和市政协的监督支持下，紧紧依靠全市人民，以奋发有为的精神状态开拓进取，以争创一流的工作理念攻坚克难，以扎实细致的优良作风狠抓落实，为全面完成今年经济社会发展各项任务、实现“十一五”发展宏伟目标、加快“科学发展、富民强市”步伐而努力奋斗！
